# Vulnerability and resilience in symbiotic interaction: a qualitative study on the lived experiences of rural older women in China

**DOI:** 10.3389/fpubh.2026.1842419

**Published:** 2026-09-24

**Authors:** Wen-Yuan Yang, Hui-Ru Chi

**Affiliations:** School of Law and Public Affairs, Jiaying University, Meizhou, China

**Keywords:** fragility, grounded theory, resilience, risk, rural older women

## Abstract

Against the strategic backdrop of comprehensively advancing rural revitalization and actively addressing population aging, rural elderly women represent a structurally vulnerable group whose living conditions and coping strategies warrant in-depth exploration. Existing studies pre-dominantly view them as passive recipients of assistance and interpret the relationship between fragility and resilience through a linear lens. Based on semi-structured in-depth interviews with 18 rural elderly women in Hakka rural areas of northeastern Guangdong, this study employs constructivist grounded theory to conduct three-level coding and develops an interactive “fragility-resilience symbiosis” model. The findings indicate first that the risks faced by this group arise from structural suppression resulting from the coupling of individual, familial, community, and cultural dimensions. The overlapping impacts of gender-based socialization, intergenerational outsourcing of maternal roles, and institutional neglect have deeply embedded fragility within local culture. Second, these women proactively cope by reconstructing cognitive frameworks, adjusting emotional support networks, and mobilizing social resources to sustain their daily lives despite structural constraints. Third, fragility and resilience are not opposing linear extremes but rather coexist and interweave amid repeated cycles of adaptation and disruption. Resilience is frequently attained at the expense of unrecognized, invisible labor. This research challenges the prevailing success-oriented narrative in resilience studies and redefines the agency boundaries of rural elderly women as constrained subjects. It advocates that relevant policies should transition from focusing solely on individual vulnerability assistance to prioritizing supportive system reconstruction, thereby offering a novel analytical framework for understanding the survival logic of vulnerable groups amidst rural social transformation.

## 1 Introduction

Global population aging is undergoing a pronounced “feminization of aging.” According to World Bank data, the global female population aged 65 years and above reached 397 million in 2020, accounting for 55% of the total population in this age group; the female population aged 60 years and above stood at 605 million and is projected to rise to 1.14 billion by 2050 (1). The United Nations Department of Economic and Social Affairs further notes that the global female population aged 60 years and above grew to 664 million in 2025 and is expected to reach 1.13 billion by 2050, representing 54% of the world's population in that age bracket (2). The World Health Organization identifies the feminization of aging as one of the core challenges within the active-aging agenda (1).

Nevertheless, longer life expectancy has not translated into equitable later-life circumstances. United Nations data suggests that old-age poverty is inherently a gendered issue. In developed countries, rising poverty in older age is entirely driven by high poverty rates among older women (3). Rooted in lifelong gender discrimination, women exhibit lower labor-force participation, earn less income, and are over-represented in informal employment. Coupled with the unequal distribution of unpaid care work, these factors leave women with far less savings and pension entitlements than men in later life (3). Notably, mainstream resilience research has long been shaped by Western individualistic psychology, which conceptualizes resilience primarily as an individual-level intrinsic trait and capacity for positive coping. This orientation depoliticizes resilience and overlooks the systemic constraints imposed on disadvantaged groups by structural inequalities of gender, age, and social class (4).

These global-scale gender-and-age inequalities are particularly salient in rural China. In the strategic context of comprehensively promoting rural revitalization and solidly promoting common prosperity, the Chinese modernization of governance in a rural aging society has become an important component of the modernization of the national governance system and capacity. According to the seventh national census of China, the percentage of elderly people aged 60 years or older in the total rural population was 23.81% in 2020. It is estimated that the percentage of the rural population aged 65 years or older will reach 35.57% by 2035 (5). The degree and speed of aging in rural areas are consistently higher than in urban areas. This is accompanied by an increasingly prominent structural feature- “gender imbalance.” In 2022, the number of rural elderly women aged 80 and above exceeded that of elderly men by 2.63 million, and this gap is projected to widen to 8.48 million by 2050 (6). For rural elderly women, research has also revealed their disadvantaged situation in the contexts of widowhood and advanced age disability (7), as well as the fact that they are significantly weaker than elderly men in dimensions such as political participation and cultural education, which leads to health problems such as loneliness (8–10). However, current studies mostly view rural elderly women as “vulnerable objects,” focusing on their passive victimization while paying insufficient attention to their subjectivity and coping wisdom in adversity (11–13). The treatment of the relationship between “vulnerability” and “resilience” is mostly confined to a linear perspective (14, 15), ignoring the dynamic attribute of their symbiotic and mutual construction.

Notably, when the perspective is lowered to Hakka areas with unique historical origins and cultural structures, relevant research is relatively scarce, and there is still a lack of systematic academic investigation into the multidimensional risk situation and coping mechanisms of Hakka rural elderly women. Hakka society is known for its clan ethics (16), gender division of labor of “men work outside, women work inside” (17), and the female labor tradition shaped by the “hard-necked” spirit (Hakka dialect: the spirit of perseverance and strength in things) (18, 19). This cultural context may both constitute local resources for rural elderly women to resist risks and exacerbate their structural dilemmas. In view of this, this study selects Chinese Hakka rural elderly women as the empirical entry point, takes the Hakka local cultural structure as an analytical field try to penetrate the mutual construction mechanism of “vulnerability and resilience,” and through the in-depth interpretation of the life experiences of 18 Hakka rural elderly women using constructivist grounded theory (20), constructs an integrated analytical framework covering four levels: individual, family, community, and culture, revealing the coupling logic between risk-generating factors and coping mechanisms.

## 2 Literature review

### 2.1 Resilience theory: from individual traits to dynamic processes

In this paper, “resilience” refers to the ability of the elderly to continue functioning properly and maintain their wellbeing in the face of negative influences (21). This is also referred to as “fortitude,” “flexibility,” and “ability to recover.” It expresses the extent to which an individual can bounce back after experiencing external stress (22). After early research focused on the individual level (23), a shift toward the strength's perspective (24) brought researchers' attention to the influence of family, community, and cultural context (25, 26). Garmezy's model of behavioral goals emphasized the role of protective factors in coping with stress and focused on the compensation of individual resources for risk (27), while Kumpfer's model of environment–individual interaction systematically integrated environmental context, individual traits, and cognitive–behavioral processes, emphasizing that resilience is a dynamic restructuring process (28). Richardson's model of mind–body–spirit balance viewed resilience as a dynamic physiological–psychological–spiritual balance and restructuring process and asserted that individuals restore balance through active restructuring after an imbalance (29).

Later research promoted the resilience concept toward a “relational” and “critical” turn. On the one hand, gerontology scholars advocate understanding resilience as a processual practice embedded in social relations and institutional arrangements rather than a steady-state ability at the individual level (4, 30). On the other hand, feminist gerontology and structural vulnerability theory point out that romanticizing resilience as a narrative of “positive coping” tends to obscure the systemic constraints imposed on vulnerable groups by gender, class, and urban-rural inequalities (31, 32). This critical perspective has key implications for this study: the “adaptation” and “resilience” of rural elderly women in adversity may be both a manifestation of subjective agency and a survival response under structural oppression (8, 33). Therefore, this study defines resilience as a procedural practice embedded in social relationships and institutional arrangements. It represents not only the ability of individuals to maintain daily order under structural constraints, but also a survival strategy fraught with tension and at the cost of self-depletion.

### 2.2 Vulnerability of rural elderly women: international and domestic academic literature

Regarding the vulnerability of rural elderly women, existing empirical studies have mainly developed along five paths. A study by the University of Washington School of Medicine revealed significant differences between rural and urban women's experiences of aging, with physical health being the primary concern, especially given the difficulties arising from limitations in healthcare (34). Traditional gender roles reinforce the practice of women taking on the majority of household tasks, and this “psychological load” continually affects sleep quality (35). Second, economic security. A study of Japan found that “bankruptcy in old age” is an increasingly common reality among elderly women in the region (36). In the United States, the wellbeing level of the elderly in rural areas is only 69% of their urban counterparts, placing them at a disadvantaged position (37). Third, mental health and social isolation. Some scholars are concerned about the high prevalence of depression and anxiety symptoms among rural elderly women (35). Relevant studies in Europe have found significant correlations among social exclusion, neighborliness, and loneliness among the elderly (38). Adequate family and interpersonal relationships play an important role in the psychological and spiritual dimensions of rural elderly women (39). Fourth, community support and environmental adaptation. The elderly have deep emotional attachments to their places of residence; however, poorly matched environmental renovations in rural areas have led this group to a “perceived mismatch” and neglected the need for elderly-friendly environments (38). Due to insufficient healthcare resources in rural communities, elderly women rely primarily on healthcare providers for medical information rather than accessing it independently through digital means (34). Fifth, cultural adaptation. Research has found that intervention measures for rural elderly female populations must take into account their cultural context and specific needs (40), such as religious beliefs, spiritual practices, and village culture. Based on the above, this article defines vulnerability as the structural disadvantageous position of rural elderly women resulting from the intersection of multiple axes, including gender, age, urban-rural structure, and cultural norms.

Regarding China‘s domestic research, relevant scholars have pointed out that the aging process in China is accompanied by accumulated risks while also harboring unique endogenous structural resources (7). However, existing literature on the cross-cutting issue of “rural elderly women” mostly stays at the descriptive presentation of vulnerability (10, 41), obscuring the empirical fact that vulnerability and resilience coexist and penetrate each other in the same subject. There is a lack of in-depth deconstruction of the institutional roots such as gender norms, intergenerational ethics, and clan structures behind them. Secondly, the investigation of resilience focuses on individual indicators at the psychological level (35), paying less attention to how relational networks, cultural practices, and daily strategies together constitute the social foundation of resilience, which easily narrativizes “resilience” as a success story and ignores the structural costs embedded within it. Moreover, empirical attention to local societies with unique cultural structures (such as Hakka areas) is particularly insufficient.

## 3 Research methodology

### 3.1 Methodological approach: constructivist grounded theory

As a systematic, procedural research methodology in social sciences, grounded theory emphasizes the bottom-up generation of data-driven theories through hierarchical abstraction and continuous comparison of empirical materials (42). This study adopts Constructivist Grounded Theory (20) as its methodological framework. Unlike the “objective discovery” stance of classical grounded theory (43), the constructivist approach ontologically acknowledges the pluralistic nature of social reality and epistemologically emphasizes that meaning is co-constructed through researcher-object interaction (44). This methodology aligns with the study's focus on elderly Hakka women in rural areas, who remain marginalized in mainstream discourse; their life experiences are deeply embedded in local cultural contexts and intergenerational relational structures, exhibiting strong contextual dependence and internal tensions.

### 3.2 Research subjects and theoretical sampling

This study adopts semi-structured in-depth interviews and on-site observation methods, and employs theoretical sampling as the basic logic for sample selection—that is, purposefully selecting subsequent cases that can enrich or test the theory based on the concepts and classifications in data analysis (20). Specifically, after identifying differentiated coping patterns among the two groups of “widowed living alone” and “intergenerational care” in the early stage of analysis, the researchers expanded the theoretical dimensions of the categories in terms of subsequent education level, living arrangement, and marital status until theoretical saturation was reached. The study pre-compiled an interview guide based on research questions and literature review, focusing on core issues such as life course, daily life practices, risk perception, and coping strategies, and flexibly adjusted according to the narrative context of respondents during the interview process.

The field sites were selected from several natural villages in the Hakka region of northeastern Guangdong Province. Choosing Hakka rural areas as the research field is not to take them as a “representative sample” of Chinese rural areas, but based on the three characteristics presented by them as a typical case. First, the profound clan tradition and highly institutionalized gender division of labor norms (16, 17). Second, the phenomenon of village “hollowing out” accompanied by large-scale labor outflow (45, 46). Third, the tension and collision between traditional ethical order and modern forces (47). The above characteristics together constitute a rich empirical field for observing the dynamic mutual construction of “vulnerability” and “resilience” among rural elderly women.

The research team initially contacted participants through a snowball effect by establishing connections with local village committees and community elders, and selected a total of 18 representative elderly women from rural areas. Their ages cover three groups: the young-old (60–70 years old), the middle-old (70–80 years old), and the oldest-old (over 80 years old). The survey was conducted in remote Hakka villages in Guangdong Province, China (all natural villages located 2–4 km from the administrative villages). Each interview lasted approximately 120 min, resulting in 32,000 characters of interview records. The interviews were audio-recorded, and all information was anonymized in accordance with the principles of informed consent, voluntary participation, and confidentiality. In-depth interviews were conducted based on textual analysis, and the interview data were coded, analyzed, and examined. The research subjects' information is shown in Table 1.

**Table 1:** Participant information.

Case no.	Age group	Educational level	Living arrangement	Marital status	Childbearing situation	Religion
**ID1**	Middle-old	Illiterate	Lives alone	Widowed	Two sons and two daughters; all settled in the city	None
**ID2**	Young-old	Junior high school	Lives with husband (Young-old) and three grandchildren	Married	Two sons, both married and working elsewhere; grandchildren remain at home for care	None
**ID3**	Young-old	Primary school	Lives with husband (Young-old)	Remarried	Two daughters and one son, all living elsewhere and seldom returning home	Buddhism
**ID4**	Middle-old	Illiterate	Lives with youngest son	Widowed	Three sons; youngest son was previously diagnosed with cancer; receives subsistence allowance	None
**ID5**	Young-old	Primary school	Separated from husband (Middle-old); lives with son and daughter-in-law	Married	Two sons and one daughter; currently living in another area to help care for grandchildren	None
**ID6**	Middle-old	Illiterate	Lives alone	Widowed	Three sons; one son passed away 2 years ago	Buddhism
**ID7**	Middle-old	Illiterate	Lives alone	Widowed	Six daughters, all settled elsewhere	None
**ID8**	Young-old	Junior high school	Lives with husband (Young-old) and two grandsons	Married	Two sons; elder son is married and working elsewhere; grandsons remain at home for care	None
**ID9**	Oldest-old	Illiterate	Lives alone	Widowed	Five sons and two daughters, all working and settled elsewhere	None
**ID10**	Oldest-old	Illiterate	Lives alone	Widowed	Three daughters, all married into other villages	None
**ID11**	Young-old	Primary school	Lives with husband (Middle-old)	Married	Two daughters and one son	None
**ID12**	Young-old	Primary school	Lives with husband (Young-old)	Married	Two sons and one daughter; daughter was adopted out at an early age	None
**ID13**	Young-old	Primary school	Lives with her son and grandson	Widowed	Two daughters; adopted one son (daughter-in-law passed away several years ago)	None
**ID14**	Young-old	Junior high school	Lives alone	Widowed	Two sons	Islam
**ID15**	Young-old	Primary school	Lives alone	Widowed	Three sons and one daughter	None
**ID16**	Young-old	Primary school	Lives with husband (Young-old)	Married	Two daughters and one son	Buddhism
**ID17**	Oldest-old	Illiterate	Lives with husband (Oldest-old)	Married	Four daughters and three sons; all settled elsewhere	None
**ID18**	Oldest-old	Illiterate	Lives with son	Widowed	Two sons; one is settled elsewhere	None

Source: created by the authors. Age group: the young-old (60–70 years old), the middle-old (70–80 years old), and the oldest-old (over 80 years old).

The above interviews strictly followed the ethical norms of social science research. The study adopted a qualitative design based on semi-structured interviews and field observations, involving no medical intervention, clinical trials, or biological sample collection, and did not require formal approval from the ethics committee in accordance with the regulations of the institution. Before data collection, the researchers fully informed all respondents of the research purpose, data usage, voluntariness of participation, and the right to withdraw at any time. Given that some elderly respondents had literacy difficulties, the researchers adopted an “oral informed consent” approach, explaining the research content item by item in Hakka dialect, and recording their willingness in the form of oral consent and audio recording after confirming understanding, reflecting cultural appropriateness and respect for the subjectivity of respondents. No personally identifiable information was collected throughout the study, all respondents were replaced by numbers (ID1—ID18) for their real names, and the original data were anonymized and used only for academic analysis, not disclosed to any third party.

### 3.3 Data transcription and analysis

Interviews were conducted in Hakka dialect, and verbatim transcription was completed by the first author (a Hakka-Mandarin bilingual) within 24 hours after the interview, forming a dialect-Mandarin bilingual compare text. Given the risk of semantic loss and cultural meaning dilution in cross-language qualitative research (48), this study drew on the suggestions of Chen and Boore (49) and Squires (50), adopting a two-stage back-translation strategy to improve meaning fidelity. **Stage 1: semantic back-translation**. A bilingual assistant who did not participate in the interview was invited to independently back-translate the Mandarin translation into Hakka expressions, and the first author compared sentence by sentence with the original recording to correct deviations caused by grammatical structure, dialect-specific vocabulary, or semantic ambiguity (49, 51). **Stage 2: contextual back-translation**. A second independent translator familiar with Hakka culture conducted contextual back-translation of key narrative paragraphs, focusing on evaluating two dimensions: first, whether emotional tone and social relationship implications were fully retained; second, whether the respondents' unique cultural metaphors (such as attributing suffering to “fate”) were presented in the translation (50, 52).

#### 3.3.1 Open coding

Open coding was conducted on the original data, during which keywords were extracted sentence by sentence and paragraph by paragraph to summarize the basic concepts and categories contained therein. A total of 98 initial concepts were generated after repeated analysis of the original interview data and open coding. After examining their differences and commonalities and removing concepts that occurred fewer than three times, 72 concepts and 28 initial categories were ultimately retained (see Table 2). For the eliminated low-frequency concepts, the researchers retained them in the form of memos as a reference for focusing on individual heterogeneous experiences in subsequent theoretical construction, avoiding the single frequency standard from obscuring marginal voices.

**Table 2:** Example of open-coding process.

Initial category	Conceptualization	Selected raw statements
Shifts in family roles (C1)	Living as a widow (01)	“My husband passed away a few years ago. Now I live alone at home and take care of everything myself.”
	Empty-nest mother (02)	“After my children grew up, they all worked away from home. I'm the only one left at home.”
	Transfer of maternal authority (03)	“After my two sons got married, they split off from us.”
Women's health predicaments (C2)	Difficulties in communicating illness (04)	“I have many health problems. I want to see a doctor in town, but I don't understand the procedures.”
	Managing the household while ill (05)	“I once fell on the mountain. I just endured the pain until my body recovered.”
	Barriers to healthcare access (06)	“It takes more than an hour on foot to reach the clinic in the market town, so I simply cannot go.”
Community mutual-aid networks (C3)	Reliance on nearby medical care (07)	“It's too troublesome to go to town. It's easier to visit the clinic here.”
	Neighbors assisting with medical help (08)	“Sometimes I ask someone who has a phone to call a doctor to come take a look.”
	Neighborhood watch and care (09)	“If the camera shows that I haven't gone out all day and I'm not answering calls, they'll ask neighbors to check on me.”
Medical financial stress (C4)	Fear of medical costs (10)	“Going to the city might cost a lot. I'm afraid I can't afford it.”
	Family savings spent on daughter-in-law (11)	“My daughter-in-law had cancer a few years ago. We spent all our savings, but she still passed away.”
	Surgery exhausting savings (12)	“I had a tumor removed last year. It cost tens of thousands of yuan—savings accumulated over many years.”
Institutional alienation of women (C5)	Women unfamiliar with health insurance (13)	“I pay for health insurance every year, but I'm not sure how it works. I only heard that it's cheaper at the township health center.”
	Alienation from administrative assistance (14)	“The village officials are stationed in another village. It's too far; no one would go find them.”
Self-repression in motherhood (C6)	Avoid burdening children (15)	“When I'm unwell, my children are working. I don't want to trouble them. They only come home during the New Year.”
Intergenerational maternal responsibilities (C7)	Declining co-residence (16)	“I don't want to live with them in the city. It's inconvenient, and I don't want to burden them.”
	Migration for childcare (17)	“My husband and I are both old. My child married late and had a baby last year. They needed help, and hiring a nanny was too costly, so I came over.”
	Relocating with grandchildren for daughter (18)	“My daughter married into this village, and there was no one to care for my grandchild, so I moved here.”
	Grandmothers as primary caregivers (19)	“My son had two boys and had to work away from home, so I take care of both grandchildren by myself.”
Wifely duties and the burden of housework (C8)	Wives upholding household labor (20)	“Cooking and laundry—these chores are all done by us women.”
	Managing home despite illness (21)	“I'm sick, but my husband can't cook, so I still have to do it.”
Mothers' multiple burdens (C9)	Overloaded work (22)	“I work during the day, then rush back to cook and take care of the children.”
	Farm work plus housework (23)	“We grow tobacco, rice, and Chinese herbs. There's never a moment to rest.”
Educational challenges in motherhood (C10)	Regret over children's education (24)	“I have no education, so I couldn't educate my children well.”
	Unable to teach grandchildren (25)	“My grandson asks me elementary school questions, but I don't know. I barely recognize any characters.”
Maternal recognition anxiety (C11)	Anxiety over being an “inadequate” mother (26)	“People often say I'm not a qualified mother.”
	Self-worth denial (27)	“I am good at nothing, like I'm useless.”
	Rural women fear the city (28)	“We rural folks don't want to go to the city—we're afraid people will laugh at us.”
Fertility-value pressure on women (C12)	Instrumentalization of fertility-value (29)	“After remarrying, I couldn't have more children. He kept scolding me for not giving him a baby.”
	Son preference pressure (30)	“My first child was a girl. The family was very upset.”
	Mother-in-law–daughter-in-law conflicts over reproductive expectations (31)	“My daughter-in-law wants a girl; I want a boy.”
Loss of wifely financial autonomy (C13)	Husband controlling wife's finances (32)	“I wanted to work to earn some money, but he wouldn't allow it and barely gave me money.”
	Husband taking wife's savings (33)	“I had saved some money, but when he drank, he would take it away.”
	Children draining mother's savings (34)	“Now the children are draining everything—my money is nearly gone.”
Fear of divorce (C14)	Fear of exiting marriage (35)	“I've never thought about divorce. At my age, what would I do after divorcing?”
	Perceived devaluation after remarriage (36)	“I'm already remarried. My first husband passed away. Being able to marry again is already fortunate.”
Support and monitoring from children (C15)	Financial support from children (37)	“The children who live away send a few hundred dollars every month; the ones nearby take care of me.”
	Emotional comfort from children (38)	“They call once a week to ask about my health. My grandchildren also call—once we start talking, it lasts a long time.”
	Remote monitoring by children (39)	“They installed cameras at the front and back doors. They check them from time to time to monitor how I'm doing.”
Resilient female survival strategies (C16)	Accepting one's lot (40)	“Life is pretty good now... but sometimes I wake up in the middle of the night, and when I think about how my life has turned out, I still feel a bit upset.
	Maternal resilience (41)	“Just go with the flow—what else can you do?”
Spiritual sustenance (C17)	Chanting scripture for peace (42)	“When I feel upset, I recite scriptures to calm my mind.”
Shrinking social connections (C18)	Disappearance of peer groups (43)	“The elders in the village are passing away one by one.”
	Diminishing neighborly ties (44)	“We barely interact.”
Emotional loneliness (C19)	Ignored by children (45)	“When I ask my children about their lives, they get impatient and say I wouldn't understand.”
	Speech dismissed as nagging (46)	“It's better not to talk at home—they say I nag.”
	Sparse parent–child contact (47)	“My children work away from home and rarely call.”
	Festive loneliness and alienation (48)	“During New Year, everyone sits together, but the young people are all on their phones, happily snatching red packets. I just sit alone watching TV, unable to join in.”
Loss of decision-making voice (C20)	Relinquishing family decision-making (49)	“Let the children decide whatever they like. I just follow their lead.”
	Being ignored in daily affairs (50)	“I'm old, so I don't really know much. I'm never consulted about anything.”
“She” alliance and mutual support (c21)	Emotional support among peers (51)	“Most of the time, I just chat with the other elders in the village or watch TV together at home. If someone isn't feeling well, we check in on each other and lend a hand when needed.”
	Close-knit neighborly support (52)	“We've lived here for decades. Most relationships are good. We help each other in times of need.”
	Going to market together with sisters-in-law and neighbors (53)	“When going to the market, I ask the elders in the village if they're going, and we go together.”
Technological barriers (C22)	Elderly women resisting new technology (54)	“My children offered to teach me (to use a phone), but I refused. What's the point for someone my age?”
	Fear of causing damage (55)	“What if I break it?”
	Struggling with smart devices (56)	“My daughter-in-law wants to buy me a smartphone that can video call and send red envelopes, but I can't figure it out.”
Technology adaptation for maternal purposes (C23)	Reliance on others for technology (57)	“The TV is so complicated. I never know how to work it and always need young people in the village to help.”
	Simplified phones for mothers (58)	“My children set up a numeric shortcut on my phone so I only need to hold down a key to call them.”
Routine and emotional closure (C24)	Daily labor routine (59)	“In the morning, I tend the vegetables in the garden. In the afternoon, when it's cooler, I go up the mountain to gather firewood.”
	Fixed entertainment routine (60)	“I really enjoy watching TV. One channel always plays dramas, so I stick to that one.”
Suppression of maternal and wifely needs (C25)	Maternal concealment of illness (61)	“I often can't sleep and feel unwell, but I don't dare say anything, afraid it'll burden my children.”
	Anxious maternal silence (62)	“I worry, but I don't say it—saying it would only add pressure on my children.”
	Anxiety about separated spouse (63)	“My husband, in his seventies, is alone at home. I feel sorry for him. I want to return and accompany him. Sometimes I cry.”
Gender socialization of women (C26)	Adopting toughness for self-protection (64)	“Back in those days, I could only protect myself and my family by being a bit tougher and more stubborn.”
	Tolerance and concession in life (65)	“My grandson was bullied at school, but I didn't know what to do; I just told him it was okay.”
Concerns about death (C27)	Awareness of approaching death (66)	“I'm 85 now, and my health is deteriorating. I don't know when my time will come.”
	Fear of frightening others (67)	“I worry that because I'm so old, if I happen to ‘pass away' in their home one night, it would scare them.”
	Choosing burial sites (68)	“As the elders pass one by one, I must choose my own resting place too.”
Maternal livelihood compensation (C28)	Elderly couples working to support the family (69)	“My husband is 5 years older and nearly 70. I sometimes help with farm work to supplement our income.”
	Willingness to support adult children (70)	“My son bought a house in the city. His income is unstable, and when he has no money, he asks us.”
	Mother repaying son's debts (71)	“My second son gambled and owed money. We gave him all our savings. Why is my life so hard?”
	Mother bearing intergenerational financial pressure (72)	“How could we possibly have money? But we can't refuse, so we grit our teeth and give.”

Source: Created by the authors.

#### 3.3.2 Axial coding

The study integrated scattered initial categories into more explanatory main categories (53). Through continuous comparison and logical classification, this study integrated 28 initial categories into two groups of seven main categories (see Table 3).

**Table 3:** Axial coding categories.

(Ai) Core category	(Bi) Main category	(Ci) Initial category	Explanation of category content	References
Risk factors (A1)	Individual dimension (B1)	Educational challenges in motherhood (C10)	Due to limited educational attainment associated with traditional gender norms, many women feel inadequate when assisting their children or grandchildren with schoolwork, which generates anxiety and a sense of regret.	(65, 66, 73, 74, 111)
		Maternal recognition anxiety (C11)	Self-doubt and diminished self-worth arising from the perception that one has not met societal or personal expectations of womanhood or motherhood.	
		Suppression of maternal and wifely needs (C25)	As both mothers and wives, women consciously suppress their own health, emotional, and personal needs to maintain family harmony and reduce burdens on family members.	
		Concerns about death (C27)	Older rural women's unique reflections on the end of life, including apprehension about the manner of death and autonomous arrangements for post-humous matters.	
		Emotional loneliness (C19)	Being marginalized in family communication, with limited emotional expression and few opportunities for empathetic listening.	
	Family dimension (B2)	Shifts in family roles (C1)	Gendered changes in family roles, authority, and space across the life course, shaped by widowhood, children's departure from home, separation of households, and other events.	(81–83, 112, 113)
		Self-repression in motherhood (C6)	Choosing silence and self-reliance when in need of help so as not to burden or trouble their children.	
		Mothers' multiple burdens (C9)	Physical and emotional overload caused by simultaneously undertaking agricultural labor, housework, childcare, and other roles.	
		Loss of wifely financial autonomy (C13)	Subordinate status in household finances, including husbands' control over women's labor income or family members' appropriation of women's personal savings.	
		Maternal livelihood compensation (C28)	Fulfilling economic maternal responsibilities by continuing agricultural labor or taking temporary work to supplement household income, even using personal savings to repay children's debts.	
	Community dimension (B3)	Shrinking social connections (C18)	Contraction of social networks due to the passing of same-age peers and declining neighborly interaction.	(86, 114–118)
		Medical financial stress (C4)	Choosing to endure illness out of fear of high medical costs, or experiencing dual financial and emotional strain when a family member suffers from a serious illness.	
		Women's health predicaments (C2)	Structural barriers to healthcare access, such as lack of knowledge and transportation difficulties, alongside gendered expectations that women continue housework despite illness.	
		Institutional alienation of women (C5)	Reluctance to seek formal support due to unfamiliarity with medical insurance policies and the perceived inaccessibility of administrative resources.	
	Cultural dimension (B4)	Wifely duties and the burden of housework (C8)	The traditional belief that “housework is a woman's duty,” which locks women into rigid roles and prevents them from stepping back from domestic labor even when ill.	(93, 94, 119–121)
		Technological barriers (C22)	Resistance or avoidance of smartphones and other digital technologies due to self-devaluation and fear of making mistakes or causing damage.	
		Fertility-value pressure on women (C12)	A woman's personal worth being reduced to her reproductive capacity, leading to blame or pressure from family or community for bearing daughters or failing to have children.	
		Fear of divorce (C14)	Reluctance to leave an unhappy marriage due to fears of being unsupported in old age and low self-worth, accompanied by a passive mindset that remarriage, if possible, would be a matter of luck.	
		Loss of decision-making voice (C20)	Being ignored in major family decisions and daily affairs, resulting in the voluntary or involuntary relinquishing of decision-making power.	
Coping behaviors (A2)	Cognitive belief reconstruction (B5)	Gender socialization (C26)	Personality traits shaped by gender norms that help women survive in harsh environments.	(9, 122)
		Resilient female survival strategies (c16)	Long-term development of a resilient life philosophy in response to persistent hardship.	
	Emotional support and adjustment (B6)	Spiritual sustenance (C17)	Elderly women seek inner peace and emotional comfort through religious practices such as chanting scriptures.	(103, 123–125)
		“She” alliance and mutual support (C21)	Informal support networks formed through chatting with female peers, going to markets together, and caring for one another.	
		Routine and emotional closure (C24)	Daily, repetitive patterns of work and leisure that create a stable yet potentially closed-off life world.	
		Intergenerational maternal responsibilities (C7)	Unpaid caregiving responsibilities, such as migration to help adult children or providing grandchild care, undertaken to support the younger generation's families.	
	Mobilization of social resources (B7)	Technology adaptation for maternal purposes (C23)	Learning technology primarily to meet maternal needs, such as communication with children, typically relying on guidance and simplification provided by children.	(126–128)
		Community mutual-aid networks (c3)	Informal healthcare support systems in the community formed around gender-based connections.	
		Support and monitoring from children (C15)	A complex interaction in which children provide both care and surveillance through financial assistance, emotional communication, and remote-monitoring technologies.	

Source: Created by the authors. The coding convention in the main text follows the pattern “core category-main category-initial category-concept sequence.”

For example, “ID1-A1-B1-C1-01” refers to respondent 1, with the core category being A1 (risk factors), the main category B1 (individual dimension), the initial category C1 (family structure changes), and the conceptualization 01 (husband death).

Risk factors unfold along the hierarchy of the ecosystem (54) and are summarized into four dimensions: the individual dimension covers categories pointing to internal psychological and cognitive states such as “motherhood education dilemma” and “motherhood identity anxiety” (55); the family dimension covers categories involving internal family power and responsibility relationships such as “family role change” and “motherhood self-repression” (56, 57); the community dimension covers resources and obstacles beyond the family boundary such as “social connection atrophy” and “female institutional alienation” (58); the cultural dimension covers categories reflecting historical gender norms such as “wife role and housework shackles” and “female reproductive value pressure” (59, 60). Coping behaviors are divided into three levels: cognitive, emotional, and behavioral according to the classic psychological framework [(61**?**, 62)], corresponding to the respondents' meaning reconstruction, emotional regulation, and practical action strategies respectively.

#### 3.3.3 Selective coding

Selective encoding aims to distill the overarching core categories and present the logical relationships between these categories through a “storyline” (53). After repeated confirmation and comparison, this study condenses the core category into “the dual coping mechanisms of rural elderly women under risk situations”: when individuals face the overlapping risks from four dimensions: individual, family, community, and culture, influenced by the tension between structural constraints and individual agency, their coping strategies exhibit the characteristics of “coexistence of resistance and compliance.” This integrates the two main categories of risk factors and coping behaviors, forming the theoretical model of this study (see Figure 1).

**Figure 1:**
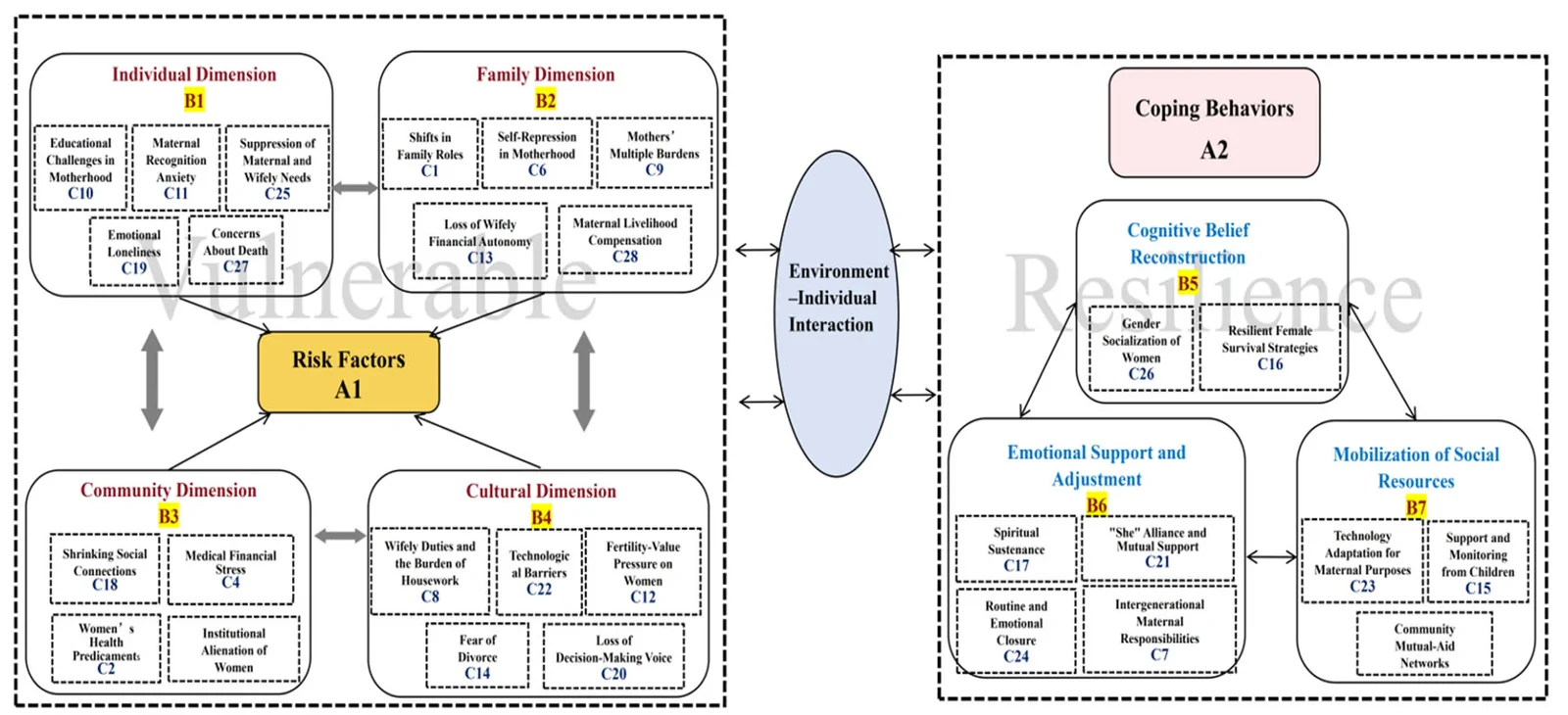
Interactive model of “fractility” and “resilience” in rural elderly women (Source: plotted in this study). The left side represents the four-dimensional risk input, the middle section depicts the interaction field between individuals and their environment, while the right side illustrates the three-tiered output process involving cognitive belief reconstruction, emotional support adaptation, and social resource mobilization. All elements are interconnected by bidirectional arrows, symbolizing continuous interaction.

The core proposition of this model emphasizes that vulnerability and resilience are two symbiotic sides of a process. Among them, four-dimensional risks (individual, family, community, culture) are regulated by triple coping strategies of cognition, emotion, and behavior in the individual interaction field, outputting a “vulnerability-resilience symbiosis” state, manifested as the coexistence of vulnerability and resilience in different dimensions (such as economic vulnerability coexisting with faith resilience), and the interweaving of adaptive wisdom and cost bearing in the same event (such as obtaining relief through the interpretive framework of “accepting fate” but suppressing one's own mental health needs). The contradictory coexistence of “bridging” (such as intergenerational connection supported by digital technology) and “rupture” (such as institutional abandonment caused by the lack of formal support) runs parallel, together constituting the dynamic characteristics of the system.

#### 3.3.4 Theoretical saturation test

The judgment of theoretical saturation follows the standards of constructivist grounded theory (20): that is, new data no longer propose substantive revisions to existing categories and their relationships (63). The researchers continuously monitored from four dimensions: new attributes, new categories, relationship conflicts, and conceptual refutations, and recorded them in memos. After completing the coding of the first 12 respondents, independent comparison of the data of the remaining six did not produce new categories or refuting evidence, so it was determined that theoretical saturation was reached. The “consistency check” by the research team further confirmed the stability of the coding process and the elevation to concepts (64).

## 4 Results and discussion

Based on the analysis and induction in this article, four dimensions including individual, family, community, and culture are summarized as the core categories of risk factors; and three dimensions including cognitive level, emotional level, and behavioral level are summarized as the categories of coping behaviors. The following two paragraphs elaborate on the two major research findings summarized through grounded theory.

### 4.1 Patterns of vulnerability among rural elderly women under multidimensional risks

Amid rapid social transformation, rural families have become increasingly smaller, more nuclear, and more spatially dispersed. As a result, the subjective wellbeing of rural elderly women has declined significantly (65). Relevant studies have pointed out that although elderly women in China have a longer average life expectancy than elderly men, they fare worse in health status, healthy life expectancy, and levels of self-care, placing them at greater risk of falling into life difficulties (66). The vulnerability of elderly women is manifested in feelings of helplessness (67), loneliness (68), and risk of depression (69). These risks arise across four dimensions—individual, family, community, and culture—and their impact is described in the following research findings. Existing literature mostly attributes the above risks to four levels: individual, family, community, and culture (9, 39, 70), but there is still a lack of systematic explanation of the coupling mechanism between the four and their symbiotic relationship with resilience, which is exactly what this study attempts to advance.

#### 4.1.1 Finding 1: the individual dimension—where self-identity and existential anxiety intertwine for rural elderly women

Self-identity refers to an individual's reflective understanding of self-based on personal experiences (71). It is the affirmation an individual with social relationships gives to their own identity and emotional orientation (72). With the rapid changes in society, the elderly are more prone to identity crises. Facing advancing age, the frustrations of intergenerational education, and the helplessness brought on by technological advancement, rural elderly women often exhibit low levels of self-recognition and self-worth. “I'm an old woman raising my grandson. He goes to kindergarten in the village, and I can manage his meals and daily needs, but when it comes to anything related to schoolwork, I just don't know what to do. I never went to school myself… sigh” *(ID8-A1-B1-C10-25)*.

The gendered binding of intergenerational bonds (73) and the diachronic extension of maternal ethics (74) reveal that the process of surrogate motherhood entails a series of costs, including time, energy, and money. These childcare costs have shifted to rural elderly women from their children's families along the gender axis, creating an “intergenerational outsourcing of maternal duties” (73).

Existing studies mostly focus on the intergenerational transfer of care labor itself (75–77). This study further finds that what is outsourced is not only labor, but also anxiety and moral responsibility.

Nevertheless, not all older women undertaking proxy motherhood experience the same intensity of anxiety. When intergenerational outsourcing of motherhood intersects with risks from other dimensions, individual-level fragility is drastically amplified. Following her son's cancer diagnosis, participant ID4 endured severe financial pressure while shouldering grandparental caregiving responsibilities: “My daughter-in-law had cancer a few years ago. We spent all our savings, yet she still passed away” *(ID4-A1-B1-C4-11)*. Unlike participant ID8′s relatively narrow “educational anxiety,” ID4′s anxiety extends beyond competence-related concerns over “whether she can properly raise her grandchildren” and escalates into an existential crisis of “being unable even to secure survival for herself and her family.” This comparison suggests that the psychological burden of proxy motherhood is not evenly distributed across older women; instead, it produces critical compounding effects when household economic-risk events occur.

Furthermore, vulnerability at the individual level presents as an internalized structural violence (78), that is, the long-term devaluation of women's educational value by society (79), which is ultimately completed through rural elderly women's self-blame for their own “ignorance” (80); and their action of persisting in surrogate motherhood in self-blame itself becomes the implicit support for the family to maintain.

#### 4.1.2 Finding 2: familial hub dimension—pressure bearing and functional compensation

The findings of the Fourth Sampling Survey on the Living Conditions of the Elderly in Urban and Rural China show that the widowhood rate among older rural women is 37.0%, significantly higher than the 16.3% observed among men (81). With the influence of multiple factors such as remoteness, living alone, and old age, rural elderly women experience higher levels of negative emotions (82). “My husband died of a sudden heart attack 2 years ago, and now I am the only one at home. In the daytime, it's fine. I go to the field to do some work. I feel very down at night. But I don't want to talk to my kids about it so they don't worry. Anyway, this is simply how life goes for me now” *(ID5-A1-B2-C1-01)*.

Rural elderly women not only shoulder multiple responsibilities but are also more likely to have their health neglected (83). In the family field, rural elderly women play the role of “risk buffer”: when offspring encounter shocks such as unemployment and illness, they absorb risks and maintain the operation of the family system through “being exploited by children,” working while sick, and intergenerational care (57).

What is more, the concrete forms of “risk-buffering cushions” vary substantially across household types. For participant ID5, buffering operates primarily through emotional self-suppression, whereas for participants ID13 and ID16, it manifests as one-way economic outflow.” Our son bought an apartment in the city. Their income is unstable, and they turn to us for money whenever they run short” *(ID13-A1-B2-C28-70)*.” How could we possibly have spare money? Yet we feel we cannot refuse, so we give despite our hardship” *(ID16-A1-B2-C28-72)*.

These two narratives suggest whether household-level “resilience” can function effectively hinges on the type of pressure borne by the risk-buffering cushion. When pressure is emotion-oriented, older women can still cope via individualized cognitive regulation. By contrast, when pressure takes the form of economic exploitation, household buffering mechanisms directly deplete their limited material resources, substantially amplifying fragility along the economic dimension. This buffer function reveals the interaction mechanism of the four dimensions: health overdraft in the physical dimension is transformed into continuous labor supply in the economic dimension, and then at the cost of self-repression in the emotional dimension, and finally is obscured in the social dimension by the discourse of “natural extension of motherhood” (80). In other words, the “resilience” of rural elderly women is built on the basis of the structural absorption and reproduction of their “vulnerability” (84). This symbiotic relationship means that understanding the plight of rural elderly women must transcend the narrative of romanticizing it as “traditional virtue” and return to the analytical framework of structural inequality (85).

#### 4.1.3 Finding 3: community interlaced dimension— relational support and institutional barriers

In contemporary China, community differentiation manifests in stark urban–rural disparities across residential environments, supporting infrastructure, governance structures, and neighborly relations (86). On one hand, peer networks continue to shrink due to mortality and migration, accelerating the erosion of the informal social capital upon which rural elderly women rely (87, 88). “One by one, the “old ghosts” (a common Hakka term affectionately used among older Peers, carrying positive connotations) have passed away. Once, I went to stay with my child for a period of time, and when I came back, I was told that the person had gone. During that time, I felt empty inside. It was hard to get used to *(ID9-A1-B3-C18-43)*.” On the other hand, the decline of informal networks failed to trigger effective supplementation by formal institutions; instead, it revealed a “service gap” between the two (89, 90). Respondents repeatedly expressed their sense of alienation from institutional resources in their accounts: “The village officials and the village committee are located in another village—too far away; who would bother to seek them out?” *(ID7-A1-B3-C5-14)*. “I pay for medical insurance annually, but I'm not entirely clear on the procedures; I've heard that seeking treatment at the township health center is cheaper” *(ID4-A1-B3-C5-13)*.

The above narratives reflect the special mechanism of the community dimension: factors such as long administrative distance, complex policy language, and passive service response make institutional resources such as medical insurance and village committees present a paradox of “visible but inaccessible,” that is, women are not uncovered by the system, but lack the cognition, information, and social relations to activate the system (91). This “institutional abandonment” is nested with the exhaustion of family, physical, and economic dimensions. When family compensation exhausts emotional reserves, physical decline weakens travel ability, and economic constraints limit help-seeking options, communities and institutions should have become the last support layer, but fail due to structural dislocation. At the same time, rural elderly women rebuild micro-support networks through grassroots methods such as neighborhood care, going to the market together, and religious practice [“If the camera sees that I haven't gone out today and can't get through on the phone, they will ask the neighbor to come and take a look.” *(ID15-A2-B7-C3-09)*], maintaining a fragile life order in the cracks of the system (92). The community thus becomes a dual site where vulnerability is amplified and resilience is self-produced.

#### 4.1.4 Finding 4: cultural collusion dimension— traditional teachings and structural exclusion

In traditional Chinese culture, the concept of “the man goes out to work while the woman looks after the house” remains deeply rooted in remote villages (93). As a result, rural women historically had fewer educational opportunities than men *(ID2-A1-B4-C12-30)*. This inequality was culturally rationalized and normalized (93). “Life used to be so difficult; surviving was already an achievement. I'm the eldest sister, and I had five younger brothers *(ID2-A1-B4-C12-30)*. We were poor, so I dropped out of elementary school after a few years and worked hard to earn money so my brothers could continue their education. Back then, everyone believed that girls didn't need much schooling, because they would get married eventually. Looking back now, I still have some grievances *(ID9-A1-B4-C20-50)*.” For elderly women in remote rural areas who are rarely exposed to changes in the outside world, traditional culture remains a prescriptive code of conduct (80). In social environments of the past, an individual who refused to accept traditional teachings would be castigated and rejected (94). It was only when they were young and in the absence of their husbands that rural elderly women could reinforce their decision-making powers and gain new spaces of mobility [(57)]. Therefore, it can be concluded that cultural dimensions possess the dual potential of both constraining and empowering.

This study reveals the “collusion mechanism between traditional discipline and modern exclusion” within this group. “The child said he wanted to teach me (using a mobile phone), but I don't want to. What's the use of an elderly person learning something” *(ID6-A1-B4-C22-54)*. The reasons why interviewees refuse to learn primarily appeal to gender and intergenerational markers such as “elderly person” and “old woman,” rather than the complexity of the technology itself.

Notably, another participant also expressed a sense of powerlessness when confronted with smartphones, yet her narrative reveals an alternative source of technological exclusion: “My daughter-in-law offered to buy me a phone for video calls and sending red envelopes, but I cannot figure out how any of these functions work” ***(ID10-A1-B4-C22-56)***. Unlike participant ID6′s account colored by “active rejection,” ID10′s predicament stems from an inability to comprehend such devices. This experience of passive predicament, rather than deliberate rejection, suggests that the effects of cultural norms in the face of technology vary across individuals.

Therefore, the path of technological exclusion is not a single-line logic of “giving up because one cannot learn,” but rather a circular structure that begins with cultural norms pre-shaping self-deprecation, leading to the rationalization of technological alienation, and ultimately promoting the reproduction of vulnerable status (95). This mechanism expands the temporal dimension of intersectionality theory (59), where the remnants of traditional discipline and the impact of modern technological changes overlap across time within the same subject, embedding vulnerability deeply within cultural structures and making it difficult to alleviate through single-dimensional interventions.

### 4.2 Resilience strategies in multidimensional interactions—active response under structural constraints

Psychological resilience, as an important ability to adapt to life's challenges, plays an essential role in helping rural elderly women cope with risks. In the model constructed for this study, three interrelated strategies emerged: cognitive belief reconstruction, emotional support adjustment, and the mobilization of social resources. The initiative and flexibility displayed by rural elderly women in their responses to risks highlight this group's autonomy and their tendency to maintain agency when facing difficulties. These characteristics are summarized in the following three findings.

It should be emphasized that the “resilience” understood in this study is not a linear success narrative of “actively coping with adversity” in the sense of positive psychology (96, 97), but a survival strategy full of tension and sometimes even self-depletion under structural constraints. The mainstream definition operationalizes resilience as a positive response to high-intensity stressors (97). Fang et al. (98) questioned that resilience should not only be regarded as an ability to adapt to challenges, it “is not always stable or positive,” and its connotation is deeply rooted in individual life course narratives and dynamically defined by social structures. This critical perspective forms a dialogue with recent research calls to break the “collusive silence” of rural elderly women and pay attention to their “resilience inequality” (13), and also provides a theoretical fulcrum for this study to reveal the processualism, contradiction, and costliness of resilience. Based on this, the following three findings aim to present how rural elderly women mobilize cognitive, emotional, and relational resources under limited conditions to maintain daily life order while bearing the unspeakable structural costs.

#### 4.2.1 Finding 5: internal interaction between personal characteristics and psychological acceptance through cognitive belief reconstruction

The cognitive control hypothesis and socioemotional selectivity theory suggest that older adults are better at employing cognitive reappraisal strategies as they age and accumulate life experience and cognitive maturity (99, 129–132). Rural elderly women draw on psychological resilience and acceptance cultivated through long-term life experiences, enabling them to reinterpret risks and strengthen their motivation for living. This inner interaction forms the psychological foundation of their coping processes and serves as a self-regulatory mechanism. Beck's cognitive model identifies negative cognitive biases, such as catastrophizing, overgeneralization, and black-and-white thinking, as detrimental to adaptive adjustment (100). However, many rural elderly women in this study demonstrated a contrasting pattern: “This is just how my body is now. I take each day as it comes—this is simply how my life will be” *(ID10-A2-B5-C16-41)*. The phrase “Let things take their natural course” often used by elderly women in rural areas is actually a form of active cognitive filtering. While acknowledging external uncontrollability, they focus their energy on specific actions that are currently within their grasp, such as planting vegetables, doing housework, and taking care of grandchildren. This dual mechanism of downward cognitive comparison and focused reduction (101). This enables them to avoid psychological exhaustion caused by persistent anxiety; “Farmland and field boundaries” have become the reproduction field of self-efficacy and identity.

The findings reveal that some participants cannot readily deploy this cognitive reappraisal strategy. Participant ID1′s narrative reflects a more bitter form of acceptance: “Life is reasonably good now… yet sometimes I wake up in the middle of the night and feel distressed thinking that this is all my life will ever amount to” *(ID1-A2-B5-C16-40)*. This persistent vacillation between resigned acceptance and inner resentment indicates that the reconstruction of cognitive beliefs is not a one-off psychological transition. Instead, it constitutes a recurrent, unstable dynamic process that may at any moment be disrupted by emotional memories.

Accordingly, among rural older women in China, the premise for this cognitive strategy to be valid is precisely the “low-expectation life script” accumulated from early educational deprivation and gender discipline (77). Their long-term position in resource scarcity and discourse margins makes “acceptance” the most feasible psychological path. This study conceptualizes it as an adaptive cognitive resource rooted in Chinese cultural beliefs (102), that is, a “cognitive weapon of the weak”—it cannot change the structural situation, but can carve out a limited and real psychological survival space for the subject in the cracks of the structure.

#### 4.2.2 Finding 6: emotional support and adjustment built a dual path of religious reliance and interpersonal connection

The emotional needs of rural elderly women are closely related to their sense of security, belonging, and self-identity. Although often viewed as “irrational,” religion has positive social functions in mitigating “existential fear” (103). Against the background of family structure changes such as children going out to work and spouse death, folk beliefs provide this group with a low-threshold and easily accessible meaning construction resource (104). As one participant noted, “In the village, including me, there are three or four older women who follow this faith. Whenever we have free time, we go to the temple to volunteer, and usually we just observe vegetarian practices and recite Buddhist scriptures” ***(ID9-A2-B6-C17-42)***.

Religious belief also reflects these women's attitudes toward life, providing spiritual comfort through devotion. Another participant explained, “I chose to follow Buddhism after my ex-husband became ill and passed away. I wondered if it was because of debts from my past life that I hadn't yet repaid” ***(ID3-A2-B6-C17-42)***.

Religious beliefs reflect their attitude toward the impermanence of life, find spiritual comfort in pious ritual practices, and re-incorporate uncontrollable traumatic events such as widowhood and illness into an understandable meaning framework through the causal narrative of “past life and present life.” This observation suggests that the effectiveness of religion as an emotion-regulation strategy hinge heavily on an individual's internal identification with its causal interpretive framework. When individuals do not endorse the core tenets of religious belief, the consolatory function of religion may break down.

Furthermore, this study finds that religious sustenance and interpersonal connection, are not two separate paths, but mutually transform and nest with each other. Common belief practices (such as vegetarianism, temple volunteer work) themselves are important media for establishing and consolidating “she alliances”—women thus not only obtain vertical connections with gods, but also weave horizontal emotional networks among women in the same village, effectively filling the emotional void formed by intergenerational separation and spouse absence (77). However, this strategy also contains its limits. When religion becomes the only outlet for comfort, it may prompt individuals to attribute structural dilemmas to personal karma rather than external conditions. Thus, it creates a certain “de-problematization” shield that obscures real problems and delays the potential for seeking institutional support and change.

#### 4.2.3 Finding 7: social resource mobilization forms a resource integration mechanism of family and community support networks

Elderly care within the family support system covers three dimensions: physical, psychological, and spatial. The rapid development of digital and intelligent technologies has opened new possibilities for improving the quality and modes of elderly care services for rural elderly women. For instance, the installation of cameras and cell phones allows for safety monitoring, emergency response, and virtual communication (105). One participant described, “When only the elderly are at home in the village, most of the younger generation (referring to our children) have installed cameras. This makes it easier for them to see what we are doing” ***(ID11-A2-B7-C15-39)***.

Through these technologies, rural elderly women can maintain more frequent and direct contact with family members living far away, transforming “physical absence” into “virtual presence.” This helps alleviate emotional emptiness and loneliness while strengthening family bonds (77).

Surprisingly, the operational logic of technology-enabled support reveals a thought-provoking structural gap. When the camera detects anomalies, the chain of substantive responses cannot be fulfilled by technology itself: “If the camera notices I have not left home all day and my phone cannot be reached, it will prompt neighbors to come and check on me” *(ID15-A2-B7-C3-09)*. The mechanism of “alerting neighbors,” rather than “automatic alarm triggering” or “remote intervention by adult children,” exposes the profound limitations of technology-enabled support. Cameras only serve a detection function, while response actions still rely on informal neighborhood networks. On the one hand, this illustrates functional differentiation between digital intermediaries and local-place intermediaries within household support systems: digital intermediaries undertake monitoring tasks, whereas local-place intermediaries perform responsive actions, and neither can function without the other. On the other hand, this reflects that mutual-aid and care constitute an important feature of rural Chinese society (106). Sayings such as “Distant relatives are not as helpful as nearby neighbors” and “Watch over each other and support each other in sickness” illustrate the strong, trust-based relationships formed through geographic proximity and social ties. These relationships effectively compensate for gaps in family support.

The unique finding of this study lies in revealing an active logic of “resource bricolage” (107): cameras are reconstructed from “monitoring devices” to “companionship media,” and smartphones are simplified into communication tools that “can hear the child's voice with one press.” This domestication process of technology reflects the creative appropriation of rural elderly women under limited resource conditions. However, this integration mechanism also contains its vulnerability. The social-support efficacy of technological intermediaries is not a stable, inherent attribute. When remote monitoring by adult children occurs without accompanying two-way communication, technology not only fails to bridge emotional distance but also amplifies the loneliness of being surveilled into a distinct source of exclusion. Its effective operation highly depends on the continuous intervention of children or young neighbors as “digital intermediaries” (such as equipment installation, operation teaching); once the intermediary chain breaks due to offspring migration or neighborhood replacement, technology transforms from an empowering tool into a new source of exclusion, making the support network swing repeatedly between bridging and rupture.

## 5 Conclusion and recommendations

After summarizing the above seven research findings belonging to the two dimensions of vulnerability and resilience and deeply analyzing their embedded psychological self-conflicts and emotional fluctuations and other turbulent evolution processes, this paper attempts to propose the theoretical contribution from linear transformation to dialectical symbiosis, extending the boundaries of existing theories; and concretely strengthening the social bottom force from the policy and practice of helping the vulnerable to reconstructing the support system; finally, **explaining the limitations of this study and future directions**.

### 5.1 Theoretical contribution: from linear transformation to dialectical symbiosis

This study focuses on the multidimensional risks faced by Hakka rural elderly women in southern China under the superimposed situation of aging, social change, and digitalization, draws on the resilience behavioral goal model proposed by Garmezy (133) and adjusts it locally, systematically analyzes the dynamic interaction between their risk factors and protective factors, and then constructs a “vulnerability-resilience symbiosis” response system, providing a new analytical perspective for understanding the elderly survival strategies under the transformation of rural society in China.

This paper sets out three research objectives at the outset: to uncover the coupling mechanisms of multidimensional risks faced by rural Hakka older women, to explore their agentic coping strategies under structural constraints, and to develop a non-linear theoretical account of the relationship between fragility and resilience. Drawing on the foregoing analyses, this study addresses these objectives along the following three dimensions.

**First, the reflection and correction of the “success narrative” tendency in resilience research** (108). Existing literature often constructs resilience as a linear, positive, and quantifiable ability improvement process, while this study, through the detailed presentation of four dimensions of economy, health, psychology, and society, reveals that vulnerability and resilience are not sequential in time, but symbiotically embedded in the same subject, the same situation, and even the same event. **Second, redefining the boundary of the subjectivity of rural elderly women, to explore agentic coping strategies under structural constraints**. They are neither “vulnerable objects” passively bearing impacts nor simple linear evolutionary subjects “from vulnerability to resilience,” but limited subjects who act after deeply recognizing structural constraints (109). **Third, vigilance against the “romanticization of suffering” narrative**. It should be particularly emphasized that the presentation of “resilience” does not mean the rationalization of the heavy labor and intergenerational sacrifices they bear (77); those daily practices repeatedly stitched between fields and stoves, temples and dialect calls, and cameras are essentially a kind of implicit contribution that has not been fully recognized by the system for a long time. This critical perspective enables a substantive analysis of the structural and institutional roots underlying the fragility-resilience nexus.

### 5.2 Practical implications: from “helping the vulnerable” to “reconstructing the support system”

Based on the analytical framework of “vulnerability-resilience symbiosis,” this study advocates that the policy thinking should shift from focusing on “helping vulnerable individuals” to “reconstructing the support system,” that is, no longer regarding rural elderly women as one-way recipients of assistance, but recognizing their agency and carrying out structural reinforcement at the weak nodes of their existing coping networks. The specific paths are as follows:

#### 5.2.1 Community level: construct a “low-threshold, high-emotion” local mutual assistance system

Considering the characteristics of Hakka rural elderly women, who exhibit resistance to complex technologies yet possess a profound need for emotional connection, it is recommended to rely on village committees and resident social workers to activate traditional Hakka clan and neighborhood mutual assistance resources such as “doing associations” and “pooling money,” and establish “peer mutual assistance groups” with Hakka dialect as the communication medium, where young-old female volunteers help the oldest-old women. The content of mutual assistance should focus on high-frequency rigid needs such as “one-click call” and “medication reminder” rather than comprehensively promoting complex intelligent devices, so as to avoid technological empowerment from being inversely alienated into a new source of exclusion.

#### 5.2.2 Cultural level: promote “digital inclusion” practices with gender reflection

The self-deprecating expression of “women don't know this” in Hakka culture is the deep cultural root of technological exclusion. In digital skills training, special sessions for women should be set up, the content should be closely linked to core concerns such as video calls and listening to Hakka folk songs, and localized encouraging words such as “don't be afraid of slowness, just be afraid of standing still” and “the more mistakes you make, the more you learn” should replace general expressions to dilute the gendered shame brought by “not being able to learn” (110).

#### 5.2.3 Policy level: promote the embedding of formal support in the “last mile.”

In response to the “institutional alienation” phenomenon revealed in the study, it is recommended to train localized “medical insurance understanders” who understand Hakka dialect and are familiar with village conditions, and village women directors, village doctors, or young-old female volunteers regularly visit households to explain the reimbursement process item by item in dialect. For rural elderly women who undertake intergenerational care and “intergenerational motherhood responsibilities,” “temporary elderly care/child care” respite services, psychological support groups, and social integration activities should be provided under the framework of rural revitalization (75 to recognize their implicit labor value in an institutionalized way.

### 5.3 Research limitations and future directions

The in-depth exploration of specific regions and groups in this study provides rich qualitative insights, but there are also several limitations. **First, the particularity of region and culture**. This study takes Hakka rural elderly women in southern China as the research object, which has limitations for research in different situations and cultural backgrounds (such as other Han rural areas, ethnic minority areas, suburban rural areas). Future research can conduct cross-regional comparative studies to test the external validity of the “vulnerability-resilience interaction model” proposed in this paper. Second, **the limitation of the time dimension**. Resilience is essentially a temporal process, and the alternation of “rupture” and “bridging” often spans a longer life cycle; this study relies on one-time in-depth interviews and is difficult to capture the in-depth trajectory of strategy evolution, which can be supplemented by life history interviews or longitudinal tracking designs in the future. Third, **the insufficiency of the intergenerational perspective**. To what extent the resilience strategies of rural elderly women are perceived and supported by their adult children, and how this intergenerational interaction in turn shapes their resilience construction, is still a direction worthy of in-depth exploration.

In the dual transformation of rural China toward deep aging and comprehensive digitalization, rural elderly women are both a group washed by the waves of the times and actors who weave survival resilience with dialects, beliefs, neighborhoods, and family affection; only when academic research, social policies, and cultural imagination jointly get rid of the single gaze of “vulnerable recipients,” recognize their implicit labor and life wisdom of stitching at the rupture and creating within limits, can the picture of rural revitalization truly accommodate the position of “her,” and let every woman who grows old between the ridges and the stove walk through the second half of life calmly on the soil of being seen, respected, and supported.

## Data Availability

The original contributions presented in the study are included in the article/supplementary material, further inquiries can be directed to the corresponding author.
